# Small molecule inhibition rescues the skeletal dysplasia phenotype of *Trpv4* mutant mice

**DOI:** 10.1172/jci.insight.182439

**Published:** 2026-01-23

**Authors:** Lisette Nevarez, Taylor K. Ismaili, Jennifer Zieba, Jorge Martin, Davis Wachtell, Derick Diaz, Jocelyn A. Ramirez, Valeria Aceves, Joshua Ito, Ryan S. Gray, David Goldstein, Sunil Sahdeo, Deborah Krakow, Daniel H. Cohn

**Affiliations:** 1 Department of Molecular, Cell and Developmental Biology, UCLA, Los Angeles, California, USA.; 2 Actio Biosciences, San Diego, California, USA.; 3 Department of Orthopaedic Surgery, UCLA, Los Angeles, California, USA.; 4 Department of Nutritional Sciences, Dell Pediatric Research Institute, The University of Texas at Austin, Dell Medical School, Austin, Texas, USA.; 5 Department of Obstetrics and Gynecology and; 6 Department of Human Genetics, UCLA, Los Angeles, California, USA.

**Keywords:** Bone biology, Cell biology, Genetics, Bone development, Calcium channels, Cartilage

## Abstract

The *TRPV4* skeletal dysplasias are characterized by short stature, short limbs with prominent large joints, and progressive scoliosis. They result from dominant missense mutations that activate the TRPV4 calcium permeable ion channel. As a platform to understand the mechanism of disease and to test the hypothesis that channel inhibition could treat these disorders, we developed a knock-in mouse that conditionally expresses the p.R594H *Trpv4* mutation. Embryonic, chondrocyte-specific induction of the mutation using *Col2a1-Cre* resulted in a skeletal dysplasia affecting the long bones, spine, and craniofacial skeletal elements, consistent with the human skeletal dysplasia phenotypes produced by *TRPV4* mutations. Cartilage growth plate histological abnormalities included disorganized proliferating chondrocyte columns and reduced hypertrophic chondrocyte development, reflecting abnormal endochondral ossification. In vivo treatment with the TRPV4-specific inhibitor GSK2798745 markedly improved the radiographic skeletal phenotype and rescued the growth plate histological abnormalities. ScRNA-Seq of chondrocyte transcripts from affected mice identified calcium-mediated effects on multiple signaling pathways as potential mechanisms underlying the defects in linear and cartilage appositional growth observed in both mutant mice and patients. These results provide preclinical evidence demonstrating TRPV4 inhibition as a rational, mechanism-based therapeutic strategy to ameliorate disease progression and severity in the *TRPV4* skeletal dysplasias.

## Abstract 2

Genetic skeletal disorders with short stature and scoliosis could be treated by a drug that inhibits the activated TRPV4 calcium channel mutated in these diseases

## Introduction

TRPV4 is a tetrameric, plasma membrane-localized, calcium permeable ion channel (1–4). The channel can be activated by a variety of environmental stimuli including low osmolarity (1, 2), heat (5, 6), and mechanical stimulation (7), as well as by both natural (8) and synthetic (9, 10) ligands. It is a member of the larger TRP ion channel family, originally discovered in the vision-impaired *trp* mutant of *Drosophila* (11), which showed transient increased potential upon light stimulation. Most TRP channels are homo- or heterotetramers, and mammalian TRP channels have been classified into 7 groups based on their structures, sequences, and functions (12, 13). TRPV4 is highly expressed in chondrocytes (14), consistent with its role in the developing skeleton, but is also expressed at lower—but functionally important—levels in many other tissues, including the bladder, neurons, lung, kidney, liver, bone, muscle, and vascular endothelium (15–26). Physiologically important TRPV4 activity among tissues with lower expression reflects the cooperative nature of channel activation, which can amplify the consequences of channel activity to boost intracellular calcium, even when there are fewer channels at the cell surface (27).

Inherited disorders due to defects in *TRPV4* were first described in 2008, when dominant *TRPV4* mutations were identified in brachyolmia (OMIM 113500), a mild short stature phenotype primarily characterized by scoliosis (14). Subsequently, heterozygosity for *TRPV4* mutations was found in several previously described skeletal dysplasias, leading to definition of a new bone dysplasia family, the *TRPV4* channelopathies (28, 29). These included, in order of increasing severity, spondylometaphyseal dysplasia Kozlowski type (SMDK; OMIM 184252), metatropic dysplasia (OMIM 156530), and lethal metatropic dysplasia (OMIM 156530), all of which have short stature with increasing deformities of the long bones and, in the nonlethal phenotypes, progressive scoliosis (30, 31). Additional disorders included within this skeletal dysplasia spectrum are spondyloepiphyseal dysplasia Maroteaux type (OMIM 184095) and parastremmatic dysplasia (OMIM 168400), which are similar to and classified with SMDK and MD, respectively (32, 33). Furthermore, a distinctive form of hand osteoarthritis (OMIM 606835; ref. 34), an inherited phenotype with isolated osteonecrosis of the femoral head (OMIM 617383; ref. 35), and a disorder with the combination of skeletal abnormalities along with giant cell lesions of the jaw and skull, and polyneuropathy (36) also result from heterozygosity for *TRPV4* mutations. Finally, a set of peripheral neuropathies, ranging from a form of Charcot-Marie-Tooth neuropathy (OMIM 606071) to a very severe form of scapuloperoneal spinal muscular atrophy (OMIM 181405), were also shown to result from dominant *TRPV4* mutations (37–40).

Among the *TRPV4* skeletal dysplasias, most mutations result in activation of the TRPV4 channel. As demonstrated by patch clamp studies and whole cell calcium imaging, primarily in transfected HEK293T cells (14, 30), the activating mutations led to increased basal (unstimulated) channel activity as well as increased response to agonists, with some mutation-specific variability in these parameters. In the transfected cells, cell-surface biotinylation studies showed that the proteins encoded by 2 of the brachyolmia mutations, p.R616Q and p.V620I, localized to the cell membrane (14), showing that mutant tetramers could be properly assembled within the cells and function at the cell surface. Studies in transfected frog oocytes demonstrate that the basal open probability among a series of 14 mutations was increased along the spectrum of clinical severity, consistent with the hypothesis that the extent of channel activation determines phenotypic severity (41).

In aggregate, the in vitro data demonstrate that TRPV4 channel activation is the primary mechanism of disease in the *TRPV4* skeletal disorders. To determine whether increased TRPV4 expression or abnormal TRPV4 activity resulting from the activating mutations produced these phenotypes, transgenic mice were constructed that overexpressed either WT or mutant TRPV4 in chondrocytes (42). Overexpression of WT TRPV4 had minimal phenotypic consequences while overexpression of p.R594H TRPV4, the most common SMDK mutation in humans, resulted in a perinatal lethal skeletal phenotype similar to lethal metatropic dysplasia. These data demonstrate that these phenotypes result from increased activity of abnormal TRPV4 but not increased expression of WT channels. The results further show that expression of the mutation in chondrocytes was sufficient to model the skeletal phenotype.

Because the *TRPV4* skeletal dysplasia mutations activate TRPV4, we sought to test the hypothesis that inhibitors of the channel would be an effective therapeutic strategy. The clinical goal of therapy would be to ameliorate the progressive axial and appendicular skeletal abnormalities that characterize these disorders. We therefore constructed heterozygous conditional knock-in (cKI) mice and established a nonlethal model of the *TRPV4* skeletal disorders by selective expression of the p.R594H *Trpv4* mutation in chondrocytes. Treatment of the affected mice with a TRPV4-specific inhibitor reduced the severity of the skeletal abnormalities, demonstrating that systemic inhibition is able to target cartilage tissue in vivo. ScRNA-Seq of chondrocyte transcripts from affected mice identified calcium-mediated effects on multiple signaling pathways as potential mechanisms underlying the defects in linear and cartilage appositional growth observed in both mutant mice and patients. Our results, thus, provide preclinical data supporting TRPV4 inhibition as a therapeutic avenue to treat the *TRPV4* skeletal disorders.

## Results

### A mouse skeletal dysplasia phenotype due to expression of activated Trpv4.

**Figure 1:**
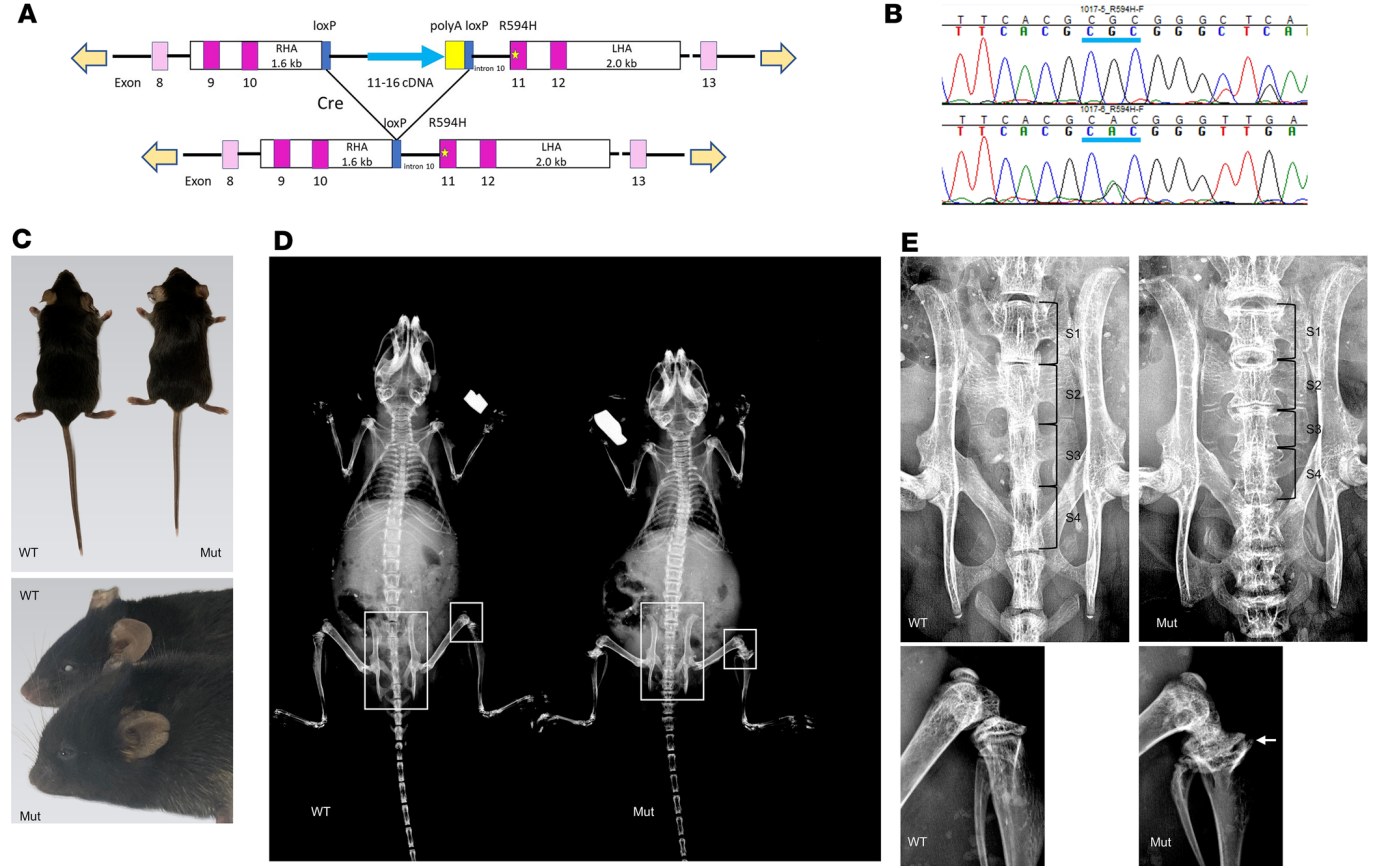
Establishment of the *Trpv4*^p.R594H^ cKI mouse model. (**A**) Diagram of the knock-in construct, which replaced one *Trpv4* allele. At the top, the structure of the unrecombined construct is shown, designed such that exons 11–16, the distal 6 coding exons, are expressed from a cDNA fragment (blue arrow). At the bottom, exposure to CRE is designed to remove the cDNA fragment to allow expression of the p.R594H mutation (yellow star) present in exon 11. (**B**) RT-PCR showing expression of the p.R594H mutation in chondrocytes from the mutant mice (central nucleotide of the underlined heterozygous mutant CAC histidine codon [bottom]) as compared with the WT CGC arginine codon (homozygous in the control at the top, heterozygous in the mutant). (**C**) Representative phenotype of *Col2a1-Cre*/*Trpv4*^p.R594H^ cKI mice at 8 weeks of age. At the top, the smaller size of the mutant (Mut, *n* = 6) relative to a control littermate (WT, *n* = 5). At the bottom, the craniofacial phenotype of the mutant (front) included a domed head and midface hypoplasia. (**D**) AP radiographs showing the generalized skeletal dysplasia of the mutant. (**E**) Zoomed in views of the areas boxed in **D**, showing platyspondyly of the identified sacral vertebral bodies as well as proximal tibiae with abnormal, wide metaphyses (arrow), relative to the WT mice.

To model the human *TRPV4* skeletal disorders in mice, our goal was to create a mouse with a physiologically relevant skeletal phenotype mild enough to allow a breeding colony to be established but severe enough to provide a therapeutic window within which to test the efficacy of channel inhibition. We initially constructed a CRISPR-mediated global knock-in mouse heterozygous for the p.V620I mutation, which produces brachyolmia in humans, but this model did not have marked skeletal abnormalities and had a lethal skeletal phenotype in the homozygous state (unpublished data). A global heterozygous knock-in for the SMDK p.R594H mutation could not be maintained because the mice had a severe, but nonlethal, skeletal phenotype (unpublished data). Consequently, we used CRISPR-enabled technology to replace 1 WT *Trpv4* allele with a cKI allele for p.R594H (Figure 1A). Mice either heterozygous or homozygous for the unrecombined cKI allele were born at expected Mendelian ratios (data not shown) and were phenotypically normal (Figure 1).

Mice heterozygous for the cKI allele were crossed with transgenic mice hemizygous for the *Col2a1*-*Cre* allele, which is selectively expressed in chondrocytes beginning at about E9.5 of mouse gestation (43). Because *Trpv4* is coexpressed with *Col2a1* in developing skeletal elements beginning at least as early as about E12.5 (44), this strategy was designed to model heterozygosity for the mutation in chondrocytes. Using RNA extracted from growth plate cartilage of E19.5 affected mice, sequence analysis of the *Trpv4* RT-PCR product revealed the presence of the mutant *Trpv4* transcript (Figure 1B), demonstrating that the conditional allele had been recombined and expressed in chondrocytes. The *Col2a1-Cre/Trpv4*^p.R594H^ mice were characterized at about 8 weeks of age, and both male and female mice were of statistically significantly smaller body length, with significantly shorter tails in females than control littermates with WT/WT, *Col2a1*-*Cre/WT*, or WT/*Trpv4*^p.R594H^ genotypes (Figure 1C and Supplemental Figure 1; supplemental material available online with this article; https://doi.org/10.1172/jci.insight.182439DS1). There were significantly shorter femur and tibia lengths in both male and female mutant mice relative to WT animals (Supplemental Figure 1). Craniofacial abnormalities included a domed skull and midface hypoplasia (Figure 1C). Radiographs of the affected mice showed axial and appendicular skeletal abnormalities that included generalized long bone shortening, platyspondyly, and an epimetaphyseal dysplasia most prominent at the proximal tibiae (Figure 1, D and E), which showed mildly flattened epiphyses and markedly widened metaphyses similar to what is observed in humans with *TRPV4* skeletal disorders.

**Figure 2:**
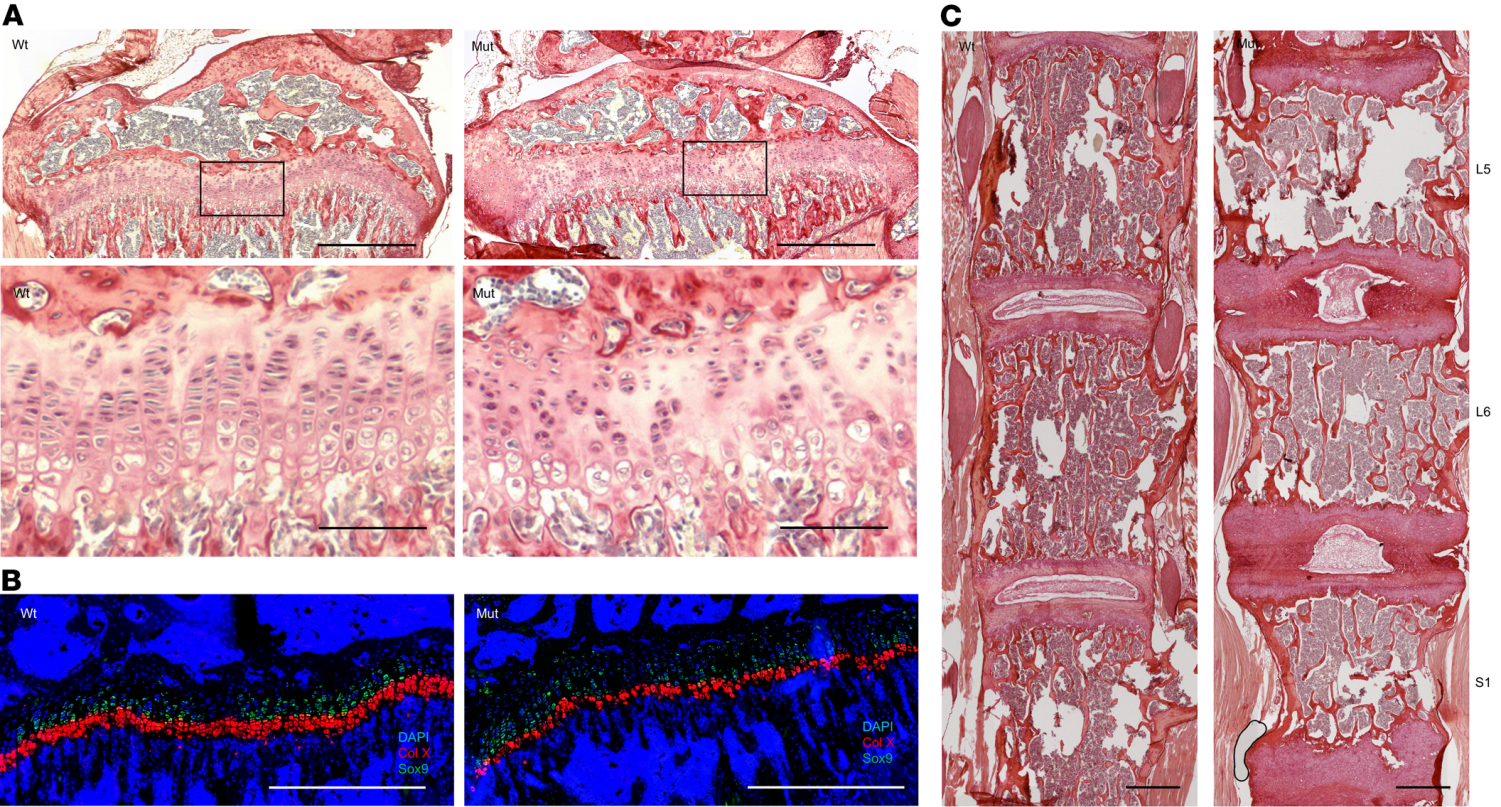
Histology of the proximal tibia and vertebral bodies in the Col2a1-Cre *Trpv4* mice. (**A**) Proximal tibial growth plates from WT (*n* = 5) and mutant (Mut, *n* = 6) 8-week-old mice stained with picrosirius red. Scale bars: 500 μm. Zoomed in (10×) views of the boxed areas illustrating the hypocellularity and abnormal column formation of proliferative chondrocytes and reduced thickness of the hypertrophic zone. Scale bars: 100 μM. (**B**) RNAscope showing expression of *Sox9* (green) in the proliferative zone and *Col10a1* (red) in the hypertrophic zone with DAPI staining (blue) identifying nuclei. Scale bars: 100 μM. (**C**) Picrosirius red–stained vertebral bodies from WT and Mut mice. Note the collapsed intervertebral disc and reduced mineralization in the mutant. Scale bars: 500 μM.

**Figure 3:**
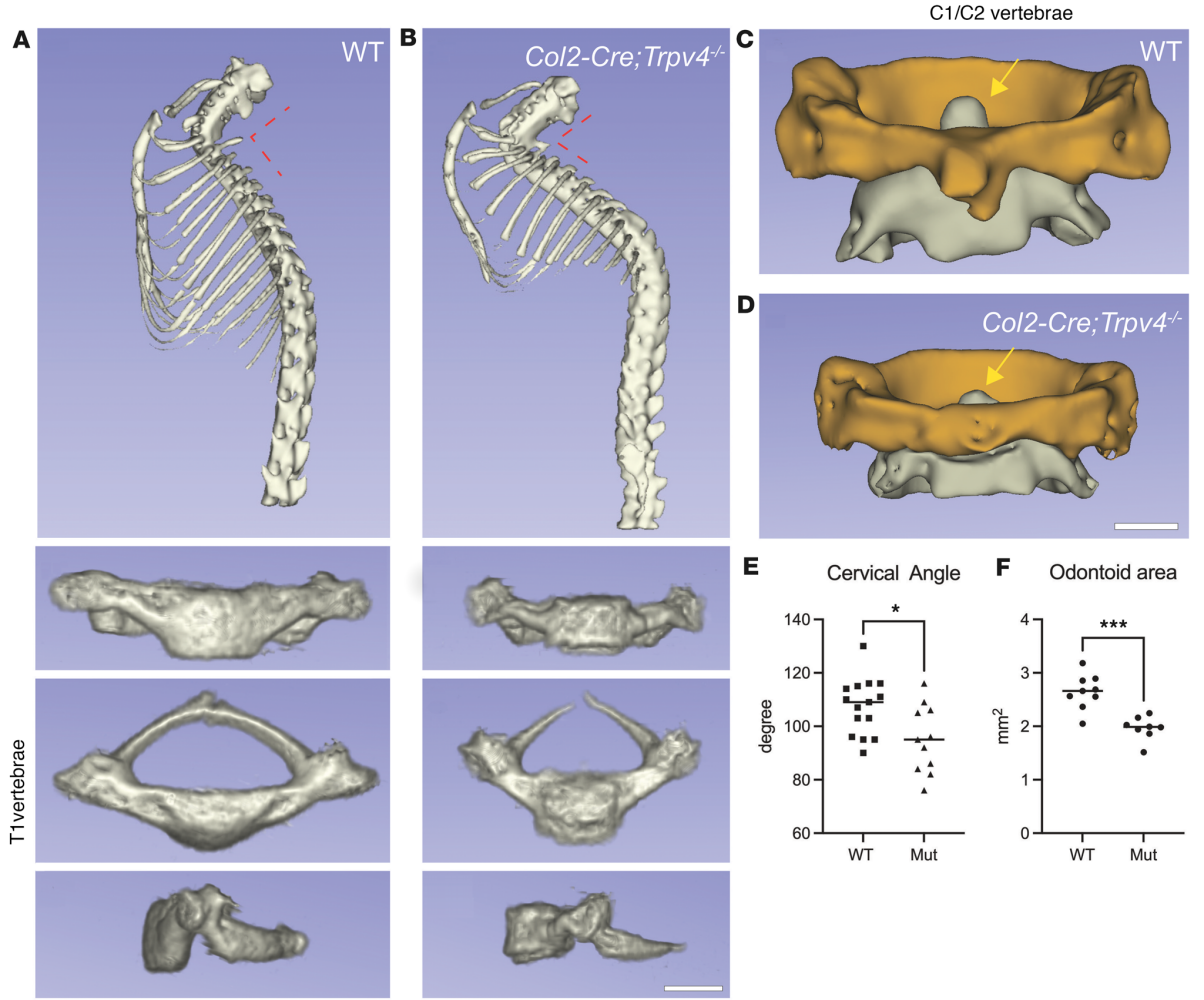
microCT of mouse atlantoaxial joint. (**A** and **B**) Reconstructed μCT images from WT (**A**, *n* = 9) and *Co2a1-Cre*/*Trpv4*^p.R59H^ mutant (**B**, *n* = 8) mice showing reduction in the cervical angle (dashed red lines). The T1 vertebral body in the mutant was smaller and poorly mineralized. (**C** and **D**) The size of the odontoid process (yellow arrows) was smaller in the mutant relative to WT mice. The C1 (atlas vertebrae) are shown in orange and the C2 (axis vertebrae) are shown in gray. (**E**) Quantitation of the reduced cervical angle in mutant mice. For this analysis, male and female mice were combined. (**F**) The circumferential area of the odontoid is shown to be statistically significantly reduced in mutant mice. Male and female mice have been combined for this analysis. Significance was determined using the Student’s *t* test (*P < 0.05, *** *P* < 0.001).

Histology of the proximal tibial growth plates (Figure 2A) in the mutant 8-week *Col2a1-Cre* *Trpv4* p.R594H–induced mice revealed loss of proliferating chondrocyte column formation in which the cells were more randomly distributed, even within individual chondrons. The disorganization of the proliferative zone and loss of chondrocyte column formation suggests that the growth abnormality primarily occurs at the level of the proliferating chondrocytes. By RNAscope, *Sox9* transcripts appeared to be normally distributed in mutant proliferative zone chondrocytes (Figure 2B), suggesting that altered regional expression of SOX9 was unlikely to be the cause of the morphological abnormality. The effect on hypertrophic cells, which were identified by position, morphology, and type X collagen expression, was more variable. In some mutant mice, the hypertrophic chondrocytes were absent, while in most others, the hypertrophic zone was reduced in mutant growth plates relative to controls (Figure 2B). Growth plate measurements reflected this variability, with no statistically significant differences in the sizes of the proliferating and hypertrophic zones or the proliferating/hypertrophic zone ratio (data not shown), relative to unaffected mice. Nevertheless, the qualitative histological data reflect the disrupted endochondral ossification observed on radiographs. Histology of the vertebral bodies (Figure 2C) also showed disruption of endochondral ossification in these axial skeletal elements, with reduction of the mineralized area of the vertebral bodies similar to that observed on radiographs, a corresponding increase in the nonmineralized intervertebral disc area, and markedly abnormal nuclei pulposi. Despite the abnormal appearance of the vertebral bodies by both histology and radiographs, in vivo μCT imaging at 8 weeks of age did not show evidence of scoliosis. However, metatropic dysplasia is often associated with odontoid hypoplasia — an underdevelopment of the odontoid process — which can compromise the stability of the atlantoaxial joint (the connection between the C1 and C2 vertebrae) and cause spinal cord compression. μCT analysis (Figure 3) did reveal a smaller odontoid in mice expressing the mutation, with a statistically significantly reduced odontoid area (Figure 3, C–F). In addition, there was an increased cervical angle observed at the boundary between the cervical and thoracic vertebrae, with both a smaller and undermineralized T1 vertebral body (Figure 3, A and B). Together, these findings show that cartilage-specific expression of the *Trpv4* p.R594H mutation is sufficient to reproduce the skeletal abnormalities seen in human *TRPV4*-related disorders. They also suggest that the phenotype arises from altered proliferation and/or differentiation of growth plate chondrocytes in both the long bones and spine.

### GSK279 systemically inhibits mutant TRPV4 in vivo.

We tested the hypothesis that in vivo TRPV4 inhibitor treatment of mice expressing the p.R594H allele in chondrocytes would ameliorate their skeletal abnormalities. Among the known TRPV4 inhibitors (45–47), we selected the GSK2798745 (GSK279) inhibitor (48) due to its specificity for TRPV4, favorable pharmacokinetic, and pharmacodynamic properties and because it had been shown to be safe in a phase I clinical trial in humans (49). In addition, structural studies have indicated that binding of the inhibitor to TRPV4 narrows the lower gate to prevent ion (calcium) entry (50) and that the *TRPV4* missense mutations do not overlap with the GSK279 inhibitor binding site (51). To determine whether GSK279 could inhibit the p.R594H mutant TRPV4 in vivo, we elected to treat postweaning *Col2a1-Cre* driven mutant mice. In addition to genotype, mutant mice at this stage could be visually recognized by their craniofacial abnormalities and smaller size relative to unaffected mice, but they did not demonstrate the marked radiographic abnormalities of the skeleton present by 8 weeks of age (Supplemental Figure 2A). However, skeletal preparations showed abnormal skeletal development characterized by a domed skull and reduced sizes of the Alizarin red–staining elements of the vertebral bodies with correspondingly increased Alcian blue staining relative to controls (Supplemental Figure 2, B and C). Proximal tibial growth plate histology revealed mild disorganization of proliferating chondrocyte column formation resulting from expression of the mutation (Supplemental Figure 2D). These results provide clear evidence of initiation of the process leading to the more severe abnormalities observed at the 8-week of age time point (Figure 1).

**Figure 4:**
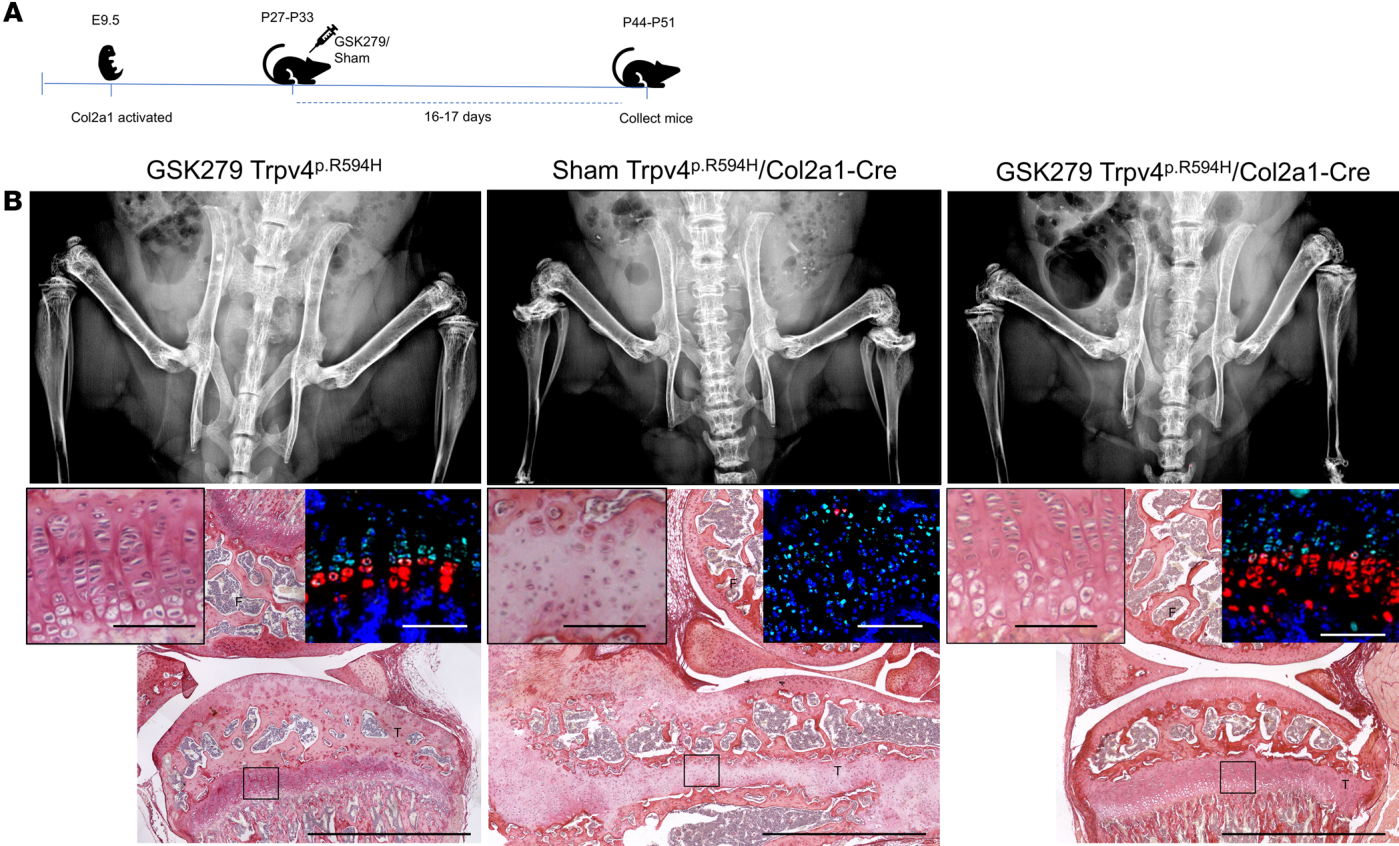
Treatment of *Col2a1-Cre*/*Trpv4*^p.R594H^ mice with GSK2798745 (GSK279). (**A**) Experimental time course in which the p.R594H mutation was activated by *Col2a1-Cre* expression during development and then treated for 17 days with GSK279. (**B**) At the top, from left to right, AP radiographs of the distal spine and knee joints of GSK279-treated *Trpv4*^p.R594H^ WT mice (*n* = 5), sham-treated (*n* = 3), and GSK279-treated *Col2a1-Cre*/*Trpv4*^p.R594H^ mutant (*n* = 5) mice. The radiographs show that the GSK279 inhibitor improved the qualitative appearance of the vertebral bodies and proximal tibiae relative to the sham-treated mice but did not fully normalize these skeletal elements. Below each radiograph are corresponding picrosirius red–stained knee joints showing the distal femur and proximal tibia. In the sham-treated *Col2a1-Cre*/*Trpv4*^p.R594H^ mice (middle panel) the marked metaphyseal dysplasia was evident with the proximal tibia (T) wrapped around the distal femur (F), and there were fewer proliferating chondrocytes that did not have the columnar arrangement present in the absence of inhibitor. There were also very few hypertrophic cells in the mutant mice. In the treated mice the knee anatomy and growth plate normalized with rescue of proliferating chondrocyte columnization and chondrocyte hypertrophy. Scale bars: 1,000 μM. For each image, there are insets showing 5× views of the proximal tibial growth plates and growth plates stained by RNAscope with probes for *Sox9* (green) and *Col10a1* (red) along with DAPI (blue), revealing that treatment also normalized expression of these molecular markers. Scale bars: 100 μM.

Male mutant mice were treated for 17 days by twice daily i.p. injections of inhibitor (Figure 4A). Neither laboratory nor veterinary personnel identified any side effects of the treatment. The treated mice had less severe skeletal abnormalities than sham-treated mutant mice, with an improved radiographic appearance of the spine and long bones, particularly the epimetaphyseal dysplasia at the proximal tibiae (Figure 4B). Notably, histology of the knee joints showed normalization of the distal femur to proximal tibia anatomy relative to sham-treated mutant mice and largely normal columnization of growth plate chondrocytes. Proliferating and hypertrophic chondrocytes, which were disorganized and/or reduced in the sham-treated mutant mice, were morphologically normal in the treated mice and normally expressed *Sox9* and *Col10a1*, respectively (Figure 4B). Thus, treatment of the mutant mice led to radiographically and morphologically improved endochondral ossification and skeletogenesis. These data indicate that the inhibitor could reach axial and appendicular growth plate chondrocytes and treat the skeletal abnormalities that would have otherwise resulted from continuing activity of the mutant channel in vivo. However, neither body, tail, nor femur lengths were statistically significantly improved and there was a significant but small increase in tibia length (Supplemental Figure 3), likely reflecting the combined effects of the mild growth plate abnormalities established prior to treatment and the short growth window available for amelioration. Despite these growth findings, the improved radiographic and histologic abnormalities support the inference that inhibition of activated mutant TRPV4 during the ongoing disease process in humans would be an effective treatment strategy, especially given the longer growth period available in humans relative to mice.

### GSK2798745 broadly inhibits activated mutant TRPV4.

**Figure 5:**
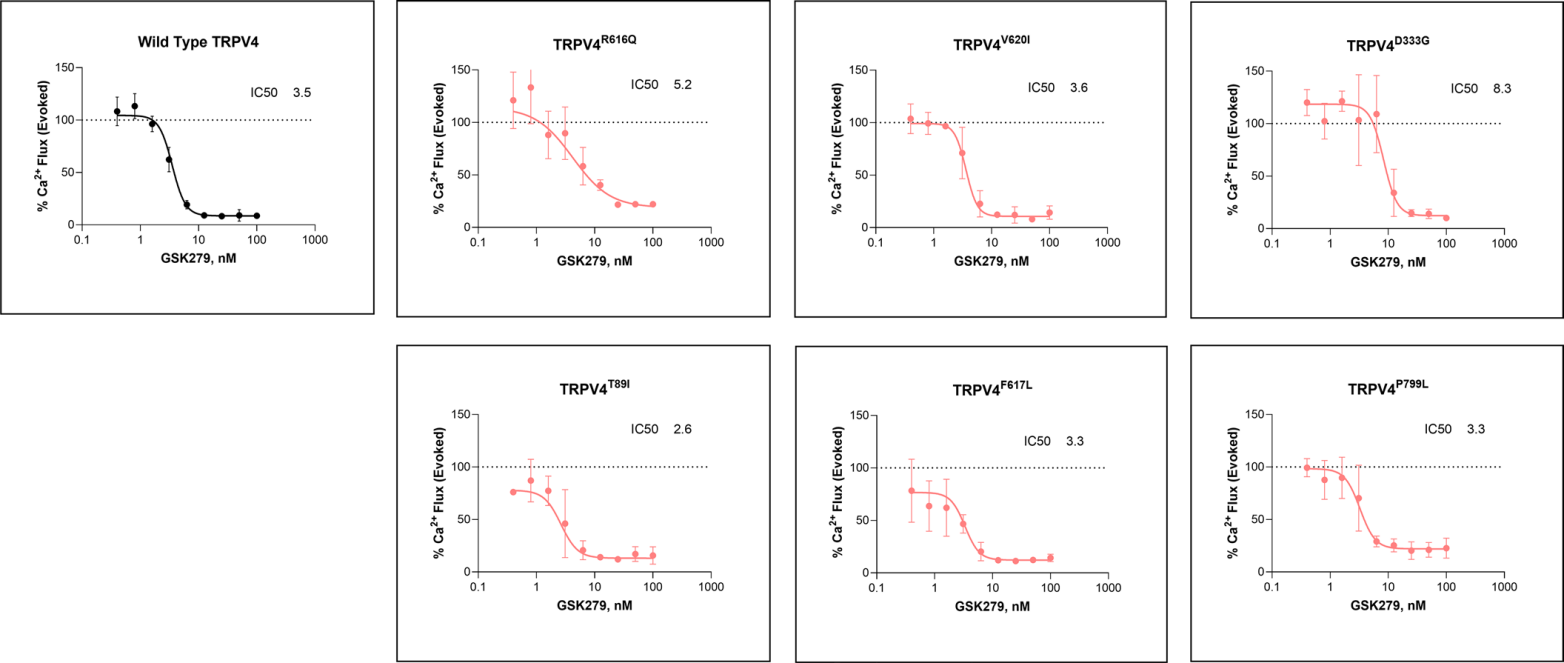
GSK2798745 broadly inhibits calcium influx in WT and gain-of-function (GOF) variants. HEK293 cells were transiently transfected with human TRPV4 WT or mutation constructs and pretreated with a range of concentrations (0.3–100 nM) of GSK2798745 (GSK279) for 1 hour prior to stimulation with 10 nM GSK1016790A (GSK101). Calcium flux was measured using a FLIPR-based calcium flux assay, and responses were normalized to the maximum GSK-101 evoked signal in the absence of inhibitor. Dose-response curves are shown for WT TRPV4 and 6 pathogenic GOF variants causal for various forms of skeletal dysplasia. Data are presented as mean ± SD of triplicates from each experiment.

Although the TRPV4 inhibitor used to treat the mice was developed as an inhibitor of WT TRPV4, the in vivo treatment data demonstrate that it was effective against the activated p.R594H mutant channel. We next sought to determine whether GSK279 could also inhibit a broader range of *TRPV4* mutations that, in humans, span the spectrum of clinical severity across the nonlethal *TRPV4* skeletal disorder phenotypes. To accomplish this, cDNA constructs with selected mutations, distributed among different functional domains of TRPV4, were transfected into HEK293T cells, and real-time whole cell calcium influx was measured using a calcium-sensitive dye. For all of the tested mutations, inhibition curves showed that there was dose-dependent GSK279 inhibition of evoked mutant channel activity, allowing IC_50_ values to be calculated for each (Figure 5). Relative to cells expressing WT TRPV4, all of the mutant channels had higher basal and evoked (agonist GSK1016790A-stimulated) (10, 41) levels of intracellular calcium that could be inhibited by GSK279 (Supplemental Table 1). These findings demonstrate that GSK279 was active against the profiled mutant TRPV4 channels and support the possibility of in vivo treatment targeted to a wide range of mutations.

### Single cell profiling identifies differentially expressed genes in mutant Trpv4 growth plates.

To identify the transcriptional consequences of expression of the activated mutant TRPV4 in the growth plate, single-cell expression data were generated from P3 WT and *Col2a1-Cre* driven *Trpv4* p.R594H growth plate cells, a time point about 2 weeks after the predicted onset of mutation expression in mutant growth plates. The single cells were generated using our previously published protocol (52), which was modified to recover a higher fraction of hypertrophic chondrocytes (see Methods). As in the prior analysis, uniform manifold approximation and projection (UMAP) cluster analysis of the RNA expression profiles identified 34 distinct cell clusters (Supplemental Figure 4A and Supplemental Data Set 1). The clusters included the 4 broad categories of chondrocytes (articular, reserve/resting, proliferating [including prehypertrophic], and hypertrophic), along with heterogeneity within each chondrocyte type. Globally, expression of the type II procollagen gene (*Col2a1*) distinguished chondrocytes from nonchondrocyte growth plate cells, most of which expressed type I procollagen (*Col1a1*) (Supplemental Figure 4, B and C). *Trpv4* was primarily expressed in the clusters corresponding to chondrocytes and thus coexpressed with *Col2a1*. The 7 articular chondrocyte clusters were all identified by expression of *Prg4*, encoding the selectively expressed articular chondrocyte protein lubricin, with subsets of articular cells distinguished by expression of sets of additional top marker genes (Supplemental Figure 4, C and D). Similarly, expression of a core set of genes (*Sox9*, *Col2a1*, *Ucma*, *Acan*, *Mgp*) identified 5 clusters of resting/reserve chondrocytes with further heterogeneity revealed by unique marker gene expression patterns (52). Four proliferating chondrocyte clusters were primarily identified by expression of genes encoding cartilage extracellular matrix proteins, and the hypertrophic chondrocytes were uniquely identified based on expression of the type X collagen gene, *Col10a1*. Eleven cell clusters corresponding to perichondrium were also identified, marked by selective expression of *Postn*, *Col1a1*, *Col3a1*, and *Prrx1*. These data validate both the cell isolation and expression profiling data as well as minimal inclusion of nongrowth plate cell types in the single cell preparations.

**Figure 6:**
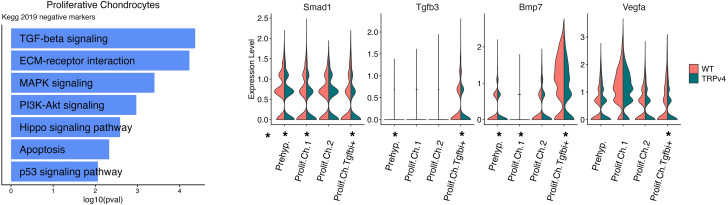
Trpv4 proliferating chondrocyte differential gene expression indicates downregulation of BMP/TGFβ signaling molecules. (**A**) Top relevant negative KEGG pathway analysis terms between WT and *Trpv4* p.R594H proliferating cell clusters. (**B**) Violin plots showing decreased expression of *Smad1*, *Tgfb3*, *Bmp*7, and *Vegfa* in 1 or more subsets of proliferating chondrocytes, quantified in Supplemental Data Set 2.

The disorganized and hypocellular proliferative zone resulting from expression of activated mutant TRPV4 suggested that there might be a profound effect of increased cytosolic calcium on gene expression and downstream signaling pathways among proliferating chondrocytes. For these cells, identified as early proliferative (cluster 14), proliferative (clusters 18,19), and prehypertrophic (cluster 10) chondrocytes (Supplemental Figure 4), Kyoto Encyclopedia of Genes and Genomes (KEGG) pathway analysis flagged decreases in multiple signaling pathways with known roles in chondrocytes. The TGF-β/BMP signaling pathway was the top chondrogenesis pathway predicted to be affected (Figure 6 and Supplemental Data Set 2), with altered expression of 15 pathway genes, including statistically significantly reduced expression of key genes (*Smad1*, *Tgfb3*, and *Bmp7*) known to contribute to chondrocyte proliferation and/or differentiation in the growth plate (53–55). These data suggest that reduced TGF-β/BMP signal transduction as a consequence of activated TRPV4 expression could contribute to the short stature observed in the *TRPV4* skeletal disorders (55, 56).

**Figure 7:**
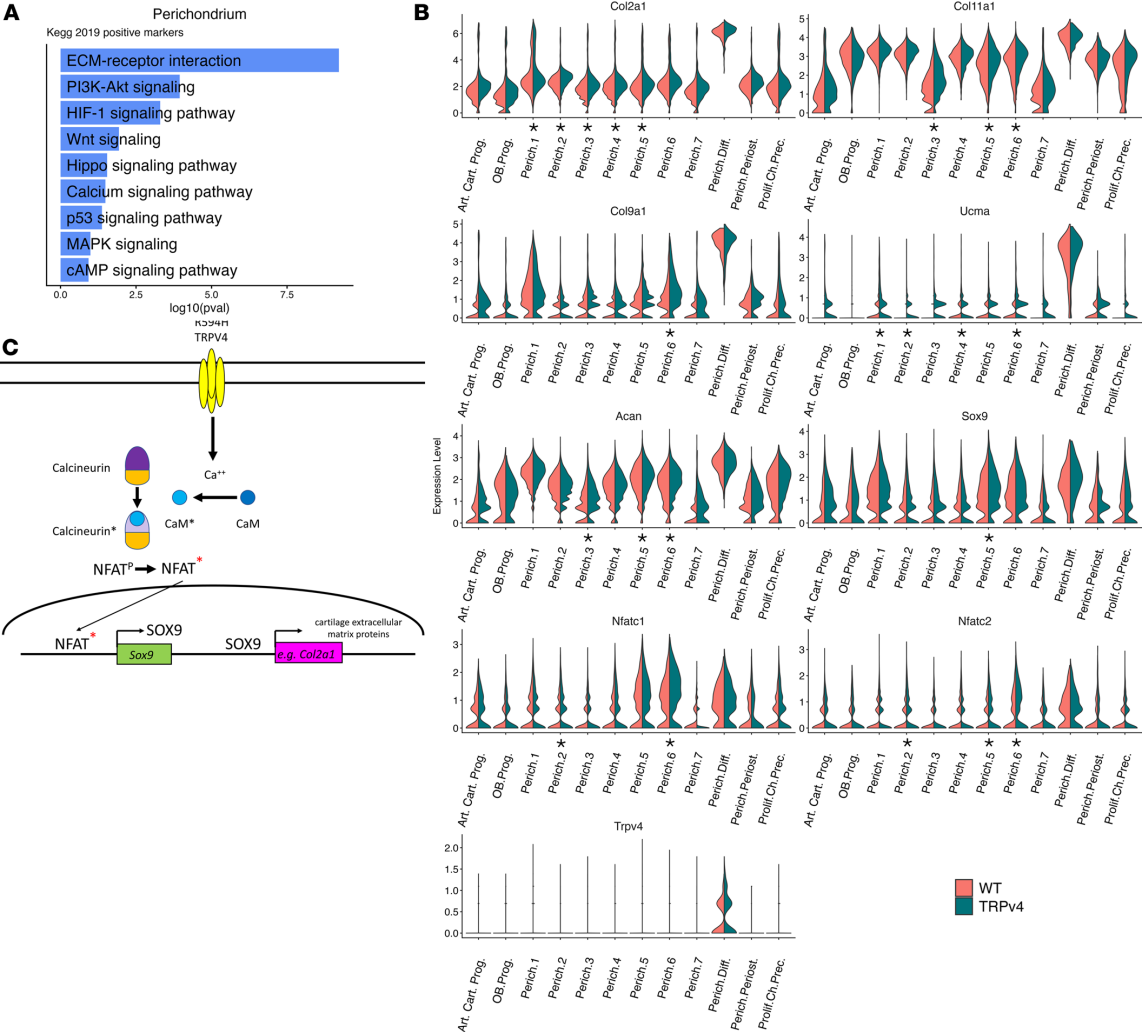
*Trpv4* perichondrial cell differential gene expression indicates upregulation of chondrocyte differentiation. (**A**) Top relevant enriched KEGG pathway analysis terms between WT and *Trpv4* p.R594H perichondrial cell clusters. (**B**) Violin plots showing increased expression of *Nfatc1*, *Nfatc2*, *Sox9*, and selected genes encoding cartilage extracellular matrix proteins, quantified in Supplemental Data Set 2. (**C**) Model of the hypothesized signaling pathway initiated by increased intracellular calcium resulting from activation of the cKI *Trpv4* allele by *Col2a1-Cre*. Under this model, increase calcium leads to calmodulin (CaM, dark blue circle) activation (light blue circle with activation indicated by the asterisk), which then binds to Calcineurin, resulting in the activation of its phosphatase activity. The activated Calcineurin then dephosphorylates NFATC1 and NFATC2, activating them and allowing them to translocate to the nucleus, where they induce *Sox9* expression. SOX9 then transcriptionally activates expression of multiple downstream cartilage extracellular matrix genes.

During endochondral ossification, cartilage appositional growth is mediated in part by recruitment of chondrocytes from perichondrial progenitors (57). Among the multiple perichondrial cell clusters distinguished in the single-cell expression analysis, the top positive KEGG pathway category identified was ECM-receptor interaction (Figure 7A). Furthermore, the most upregulated genes in the mutation-expressing perichondrial cells exhibited about a 50% increase in expression of genes encoding the cartilage collagens (types II, IX, and XI) as well as additional genes encoding cartilage extracellular matrix proteins known to be selectively expressed in chondrocytes (e.g., *Ucma*, *Acan*) (Figure 7B and Supplemental Data Set 2). These upregulated genes are all positively regulated by *Sox9* (58), which was similarly upregulated. Increased expression of *Nfatc1* and *Nfatc2*, upstream positive transcriptional regulators of *Sox9* (59, 60), was also observed, consistent with activation of the calmodulin/calcineurin signaling pathway. *Nfatc1* and *Nfatc2* are themselves activated by increased intracellular calcium (61), a predicted consequence of the expression of mutant, activated TRPV4. The upregulation of cartilage extracellular matrix protein genes is compatible with the enlarged joints observed in the mutant mice and in patients with *TRPV4* skeletal disorders, and these findings suggest that one consequence of TRPV4 activation in perichondrially derived chondrocytes may be increased chondrogenesis with abundant extracellular matrix gene/protein expression, especially in the knees and hands.

## Discussion

This study describes establishment of a conditional, nonlethal, heterozygous mouse model for the *TRPV4* skeletal disorders that is amenable to testing the effectiveness of treatment modalities aimed at inhibiting the activated TRPV4 that produces this spectrum of skeletal dysplasia phenotypes. Embryonic activation of the mutation resulted in a phenotype at 8 weeks of short limbs, abnormal vertebrae, and craniofacial abnormalities consistent with the human *TRPV4* skeletal dysplasias. The timing of induction of the mutation was consistent with the normal developmental timing and cartilage-selectivity of *Trpv4* expression in the skeletal elements, where it is coexpressed with type II procollagen (44). Growth plate histology at the 8-week time point was markedly abnormal, with disorganized and sparse proliferating chondrocytes as well as a smaller hypertrophic zone, consistent with the abnormalities in skeletal development observed in the mice. We speculate that the reduced number of proliferating growth plate chondrocytes available for further differentiation resulted in the observed reduction in morphologically identifiable hypertrophic chondrocytes expressing type X collagen in most of the mutant mice.

We tested the hypothesis that in vivo administration of the GSK279 TRPV4 inhibitor would improve the skeletal phenotype of the heterozygous cKI mutant mice. Because *Col2a1*-*Cre* expression begins at E9.5 (43), and our data demonstrate *Col2a1*-*Cre* mediated expression of *Trpv4* p.R594H in cartilage by at least as early as E19.5, this experiment models developmental expression of mutant TRPV4 in chondrocytes. Early induction of the mutation is important in a human context because individuals heterozygous for *TRPV4* mutations that produce SMDK and MD have skeletal abnormalities that can be identified at birth. The histological abnormalities of growth plate chondrocytes evident by P21 (Figure 3), provide a functional readout for the consequences of embryonic expression of the mutation on skeletogenesis. In the mutant mice, the improvement in the radiographic skeletal phenotype upon inhibitor treatment, along with dramatic correction of the organization of growth plate chondrocytes, provides strong evidence that the systemic treatment was effective. This in turn suggests that intervention after the disease process has been established but, while there is still a substantial time remaining for growth, could ameliorate the skeletal phenotype in individuals with *TRPV4* skeletal disorders. We expect that early intervention, prior to the development of major skeletal abnormalities, would provide the widest window for effective treatment and be the most helpful in preventing the bone deformities that would otherwise arise.

The phenotype of *Trpv4*–global KO mice (62) provides additional context for the potential consequences of TRPV4 inhibition. *Trpv4*-KO mice were born at normal Mendelian ratios and appeared to be behaviorally and physiologically normal (62). This suggests that either TRPV4 activity is dispensable or that, in the context of absence of TRPV4, there are other mechanisms used to achieve or approximate intracellular calcium homeostasis during growth. Alternative pathways, if present, might compensate for abrogation of the known function of TRPV4 in the skeleton as well as the many nonskeletal tissues in which TRPV4 activity is important, perhaps explaining the absence of overt abnormalities in most organ systems in the *Trpv4*-KO mice. With use of appropriate Cre drivers, using our cKI mouse model, more detailed tissue-selective effects of mutant TRPV4 activation could be explored in nonskeletal tissues.

In the *TRPV4* skeletal disorder spectrum, the main findings in the appendicular skeleton are short stature and prominence of the large joints with limited joint mobility. The *TRPV4* mutations result in increased channel activity, with the extent of channel activation correlated with clinical severity (63), but the consequences of the resulting increase in intracellular chondrocyte calcium have not been extensively explored. In the cKI mice described in this report, the growth plate histology demonstrates gross disorganization of the proliferative zone, with lack of chondrocyte column formation and sparse proliferating chondrocytes by the time the cKI mice reached 8 weeks of age. These data suggest that the short stature in the *TRPV4* skeletal disorders primarily results from proliferating chondrocyte abnormalities. The single-cell expression analysis supports this inference, suggesting that downregulation of the TGF-β/BMP signaling pathway, which could reduce chondrogenesis via the combined effects of decreased proliferation and reduced hypertrophic chondrocyte differentiation, contributes to the phenotype (64, 65). Thus, these data are compatible with a previously suggested role for reduced BMP signaling activity as a component of reduced long bone growth in the *TRPV4* skeletal disorders (66, 67).

The prominent joints observed clinically in the *TRPV4* skeletal disorders suggest abundant chondrogenesis at these anatomic locations. The limited available human histology (31, 68, 69) is consistent with increased cartilage appositional growth, a developmental process mediated in part by differentiation of chondrocytes from perichondrial progenitors (70). Our differential single-cell gene expression data for mutant perichondrial cells demonstrated upregulation of mRNA encoding all of the major cartilage extracellular matrix proteins as well as *Sox9*, the upstream regulator of these genes (58). In addition, the *Nfatc1* and *Nfatc2* transcription factor genes, which positively regulate *Sox9* gene expression (59, 60), were also upregulated. Since the NFAT genes are induced by increased intracellular calcium (61), which is a predicted result of expression of mutant TRPV4, these data are consistent with a gene expression pathway that could explain the differential gene expression result. In this model (Figure 7C), as perichondrial progenitors are recruited to differentiate into chondrocytes, they would begin to express *Col2a1*, activating the *Col2a1-Cre* allele to recombine the cKI construct so that it would express the mutant TRPV4 allele. *Trpv4* expression, including both the mutant and WT alleles, reflecting heterozygosity for the mutation, would then occur as the normal chondrocyte gene expression program is activated in the differentiating perichondrial chondrocyte progenitors (Figure 7C) (71). The resulting increase in intracellular calcium, primarily due to expression of mutant TRPV4, would then activate the calmodulin/calcineurin pathway, leading to upregulation of the NFAT transcription factors (72). The increased transcriptional activity would predictably lead to a further enhancement of *Sox9* expression and additional increased expression of cartilage extracellular matrix proteins, reinforcing and exacerbating the consequences of TRPV4 activation. Under this model, the increased cartilage matrix protein gene expression, possibly but not necessarily along with increased recruitment of perichondrial progenitors to chondrocytes, would lead to the observed joint enlargement. Perichondrial *Cre* drivers could be used to further test this hypothesis using the mouse model described here.

Despite the suggested mechanistic clues gleaned from the single-cell gene expression data, our insights are necessarily limited. First, we selected the P3 time point, which was about 2 weeks after the expected onset of expression of the mutation, and at a stage when histological abnormalities in the growth plate were just beginning to be visible but before the disease process became more advanced. At later stages, when the radiographic and histologic anomalies were more apparent, similar data could more clearly reflect the ongoing disease process. Second, there is known, biologically relevant crosstalk among growth plate signaling pathways (73), and additional altered pathways beyond TGF-β/BMP signaling were identified in the scRNA-Seq experiment (Figure 5). Thus, further experimental validation would be required to establish the hierarchy of disrupted signaling implied by the data. And third, the contribution of altered expression of the proteins derived from the changes in gene expression, both individually and collectively, have not been assessed in this study.

The mutations that produce the *TRPV4* skeletal disorders are widely distributed across the protein and localized different TRPV4 functional domains. These data, along with the highly variable responses of the different mutations to agonists, support the hypothesis that the mechanisms that increase channel activity could vary among mutations. For example, mutations adjacent to the calmodulin binding site are known to lead to a poor response to calcium-mediated channel activation, while more distant mutations respond well (63). In addition, the downstream consequences of increased intracellular calcium in chondrocytes are complex (74), and data supported by the single-cell gene expression results presented here suggest potential dysregulation of many signaling pathways in growth plate chondrocytes that express mutant TRPV4. Thus, therapeutic targeting of 1 or more of the downstream molecules and/or pathways may not be either a confident strategic approach or result in a completely effective therapy. For this reason, global inhibition of TRPV4 activity, as demonstrated here for GSK279, may be a preferred treatment avenue. Despite the safety of GSK279 in a phase 1 clinical trial in humans, 62% of the GSK279 dose yielded a metabolite with lower inhibitory activity and other unfavorable physicochemical properties that rendered the metabolite less effective than the parent molecule (75) and, therefore, of lesser value for treating people. Thus, while our data provide proof of principle that systemic TRPV4 inhibitor therapy would be the most useful approach to treatment of the *TRPV4* skeletal disorders, inhibitors with improved activity will be needed to identify small molecules that are more clinically efficacious.

## Methods

### Sex as a biological variable.

For animal experiments, both male and female mice were studied. Although qualitative measures did not vary with sex, the larger size of the male mice precluded combining male and female animals for some quantitative analyses, mainly skeletal element measurements. Where male and female data do not differ, male and female data could be combined. Finally, in some cases, depending on breeding yield, some experiments used only male or only female mice.

### Animals.

Mice were bred in the Department of Laboratory Animal Medicine (DLAM) at UCLA. Ad libitum access to food and water were provided in temperature-controlled rooms. Daily health checks were provided by laboratory and DLAM personnel. Extraskeletal side effects in either the untreated or treated mice were not observed. Strains used were B6.Cg-Acan^tm1(cre/ERT2)Crm/J^ (Jackson Laboratory, 019148) (76); B6;SJL-Tg(*Col2a1-cre*)1Bhr/J (Jackson Laboratory, 003554) (43), which was generated on a B6SJLF1 background and then crossed to C57BL/6J twice; and the *Trpv4* p.R594H–cKI strain described in this report, which was generated at the Jackson Laboratory on a C57BL/6J background. For the inhibitor studies, the GSK2798745 inhibitor (MedChemExpress #HY-19765) was administered by twice daily IP injection at 10 mg/kg for 14–17 days.

### Radiography.

X-rays were taken using a Faxitron model LX-60 cabinet machine according to the manufacturer’s protocol. High-resolution μCT images were obtained using Quantum GX2 machine (Perkin Elmer) with a 0.2 mm Cu filter at settings of 90 kV voltage, 88 μA current, and a voxel size of 72 μm. From the original μCT scans, the neck region (i.e., the base of the skull and vertebrae C1–C7) was segmented as a volume of interest (VOI) using Bruker CTAn (Bruker). This VOI was then imported into 3D Slicer to reconstruct a segmentation of C1 and C2, including the odontoid process.

### Histology.

Knee joints containing the distal femoral and proximal tibial growth plates were dissected, fixed for 24 hours in PFA, decalcified and paraffin embedded. Sections at 5 μm were prepared, deparaffinized, and stained with picrosirius red. Growth plate zones were measured using Fiji. The average of 3 measurements were calculated from each slide. The selected slides showed the Grooves of Ranvier at the margins and growth plates were measured in the middle and at the 2 halfway points from the middle to the edge of the secondary ossification center.

### RNAscope.

We performed RNAscope analysis using a previously published protocol (52). Briefly, we used growth plate samples fixed in 4% paraformaldehyde overnight followed by paraffin embedding. Cartilage sections were probed using the RNAscope Multiplex Fluorescent V2 assay from ACDBio according to the manufacturer’s recommendations. The standard protocol was followed with some modifications specific to cartilage samples. Prior to deparaffinization, slides were baked at 60°C for 2 hours to ensure that the cartilage tissue adhered to the slides. Following deparaffinization, slides were again baked at 60°C for 30 minutes. Additionally, the protease treatment step was replaced with incubation using a custom reagent provided by ACDBio for 15 minutes at room temperature (RT). RNAscope-processed slides were imaged at 20× original magnification using the Echo Revolution microscope, and images were processed using Adobe Photoshop. Probes used were *Sox9* (Catalog #401051), and *Col10a1* (Catalog #467961).

### Transient expression of Trpv4 skeletal dysplasia mutants in HEK293 cells.

Prior to transfection, HEK293 cells were cultured in a 37°C, 5% CO_2_ incubator and grown to ~80%–90% confluency. The day before the calcium experiment, reverse transfections were performed in 384 well black/clear plates and incubated at 37°C, 5% CO_2_ overnight. All reagents were equilibrated to RT prior to transfection. The number of macrotubes (Alkali Scientific, C2520) corresponded to the number of transfections (1 tube = 1 mutant). For 150 assay wells, macrotubes were incubated for 20 minutes at RT with the transfection mixture. Each tube contained 1.25 mL OptiMEM (Gibco, 31985062), 7.5 μg hTRPV4 Mutant cDNA or 5.6 μg hTRPV4 WT cDNA, and transfection reagent. The transfection reagent, FUGENE 6 (Promega, E2692), was added so the final ratio in the macrotube was 6 μL FUGENE 6 per 1 μg cDNA. During the cDNA:FUGENE 6 incubation, cells were lifted from the flask with TrypLE (CAT# 12604013) and centrifuged for 5 minutes at 1K RPM. The cell pellet was resuspended to 1M/mL with fresh culture media (1X DMEM; Gibco, 11965092), 10% heat inactivated FBS (Gibco, 16140071), 1x Pen-strep (Gibco, 15070063). Upon completion of the 20-minute incubation, 2.5 mL of cell suspension was added to each macrotube and inverted for thorough mixing. This mixture was plated so each well contained a final volume of 25 μL in a black/clear-bottom 384 well plate (Corning, 3764). To minimize edge effects, the outer wells of the plate were filled with at least 50 μL PBS. The plate was centrifuged for 1 minute at 1K RPM before placing the plate in a 37°C, 5% CO_2_ incubator overnight.

### Assessment of calcium flux in TRPV4 mutants.

Calcium flux was measured using the FLIPR Calcium 6 Assay Kit (Molecular Devices, R8191). The day of the experiment, fresh probenecid was added to the Calcium 6 dye stock at a final concentration of 5 mM. Each assay well received 25 μL of Calcium 6 dye, followed by subsequent addition of compound with the Multidrop Pico 8 Digital Dispenser (Thermo, 5840500). To ensure that all reagents were thoroughly mixed, the plate was centrifuged for 1 minute at 1K RPM and incubated for 1 hour at 37°C, 5% CO_2_. Instead of Component B, an HBSS Solution (20 mM HEPES [Gibco, 15630080], 1X HBSS Buffer [Gibco, 10010-023]) was prepared to reconstitute the vials. Water-soluble probenecid was purchased from Invitrogen (CAT #P36400). During the 1-hour dye incubation, an intermediate dilution plate was prepared with 6X 10 nM GSK101 or vehicle in HBSS solution and placed inside the FLIPR Penta. Upon completion of the incubation, the dye-loaded assay plate was equilibrated to RT and positioned inside the Penta. A 30-second baseline read was obtained before transferring 10 μL of agonist from the intermediate plate to the assay plate. Changes in RFU were recorded for an additional 180 seconds before the data were exported, with the following parameters: Average RFU 1–30 seconds and Maximum RFU 35–210 seconds. The GSK1016790A (GSK101) activator was from MedChemExpress, LLC (CAT #HY-19608, CAS: 942206-85-1). The GSK2798745 (GSK279) inhibitor was from MedChemExpress, LLC (CAT #HY-19765, CAS: 1419609-94-1).

### Single cell RNA-Seq.

The knee joints of 2 WT and 3 *Col2a1-Cre* driven *Trpv4* p.R594H P3 mutant mice (all littermates) were separately isolated; dissected free of tendon, ligament, and other nongrowth plate tissue; and digested for 45 minutes at 37°C with 0.1% collagenase type I (Gibco) to remove any remaining adherent tissue. Single cells were obtained by removing the growth plates at the bone-cartilage junction and digesting at 37°C with 0.1% collagenase type II, 0.5% trypsin (Gibco). The cell suspension was strained through a 0.7 μM filter to remove undigested tissue, rinsed with cold PBS, and the cells collected by gentle centrifugation at 300*g* for 5 minutes at 4°C and resuspended in 0.5 mL cold PBS. Viability was determined by trypan blue exclusion and the size profile of the resulting cells determined using a Countess Cell Counter (Thermo Fisher). In total, 40,000 single cells per sample were isolated using cartridges from the BD Rhapsody HT Express Single Cell Analysis System in the UCLA Technology Center for Genomics and Bioinformatics Core, according to the manufacturer’s recommendations. Imaging of the captured cells in the cartridges confirmed that the cell size distribution mirrored the input sizes. Cell lysis and barcoding used the manufacturer’s protocol and bidirectional 100 bp sequence analysis of 20,000 cells per sample with 50,000 reads per cell was generated using a NovaSeq S4 sequencer. The Seven Bridges pipeline was used for initial data processing to demultiplex, assign barcodes, and quantify transcripts. Downstream analyses were similar to those described in Zieba et al. (52). Briefly, using Seurat V4, cells with less than 200 expressed genes, more than 6,000 expressed genes, or over 10% mitochondrial transcripts were excluded prior to data normalization and integration using the SCTransform method. The normalized counts were used for clustering (UMAP) and to generate differential gene expression profiles.

### Statistics.

Growth plate data were acquired using ImageJ (NIH) and analyzed using a Mann-Whitney *U* test to determine significance. Data represent mean ± SEM. scRNA-Seq data were analyzed, visualized, and statistically compared using R and the Seurat V4 package. *P* < 0.05 was considered significant. Growth data throughout were assessed using the 2-tailed Student’s *t*-test.

### Study approval.

All mouse studies were performed according to AAALAC standards under an IACUC approved protocol reviewed by the UCLA Animal Research Committee.

### Data availability.

All of the data from this study are presented in the article Supplemental Data, Supporting Data Values file, and public repositories. All materials, are freely available upon completion of a material transfer agreement with UCLA and requests can be directed to the corresponding author. The cKI mice are available from the Jackson Laboratory under the identifier JAX #040352 C57BL/6J-*Trpv4^em1Dhco^*/J). scRNA-Seq data were deposited into the NCBI Gene Expression Omnibus (GEO) under accession no. GSE263775. Supporting analytic code can be accessed on GitHub (https://github.com/jenzieba/Cartilage_ScRNAseq; commit ID c79e0b9).

## Author contributions

DHC, DK, DG, and SS conceptualized and designed the study. TKI, JZ, JM, DW, DD, and JAR conducted the experiments. JZ processed and both JZ and DHC analyzed the single-cell data. VA, JI, and RSG analyzed the μCT data. DHC and LN wrote the original draft of the manuscript. All authors reviewed and edited subsequent drafts the manuscript and approved the final product.

## Funding support

- Actio Biosciences
- Luskin Orthopaedic Institute for Children and the Orthopaedic Hospital Research Center
- Contributions from the O’Neal family.

## Supplementary Material

Supplemental data

Supplemental data set 1

Supplemental data set 2

Supporting data values

## Version 1 01/23/2026

Electronic publication
