# Supplementary material for: Small molecule inhibition rescues the skeletal dysplasia phenotype of Trpv4 mutant mice
Source: JCI Insight. 2026 Jan 23;11(2):e182439. doi: 10.1172/jci.insight.182439 (PMC12892883; doi:10.1172/jci.insight.182439)
Supplement: Supplemental data [file jciinsight-11-182439-s086.pdf]

Supplement

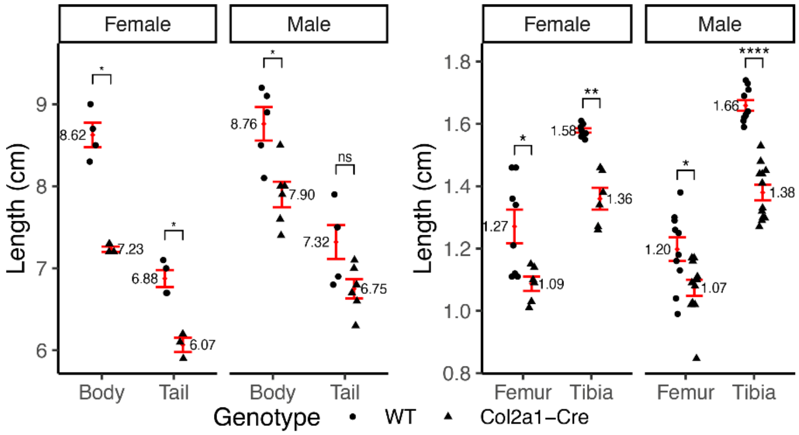

Supplemental Figure 1. Sizes of *Col2a1-Cre Trpv4*$^{\mathbf{p.R594H}}$ female and male mice compared with WT controls. (**Left**) Average body and tail lengths in mutant mice (n=3 for females, n=6 for males) were significantly smaller compared to unaffected mice (n=4 for females, n=5 for males). (**Right**) Average long bone lengths (femur and tibia) were significantly smaller in mutant mice compared to unaffected mice. P values were calculated using a Mann-Whitney *U* test. Error bars indicate mean ± SEM. Significance of the differences: ns=p>0.05; *=p<0.05; **=p<0.01; ***=p<0.001; ****=p<0.0001).

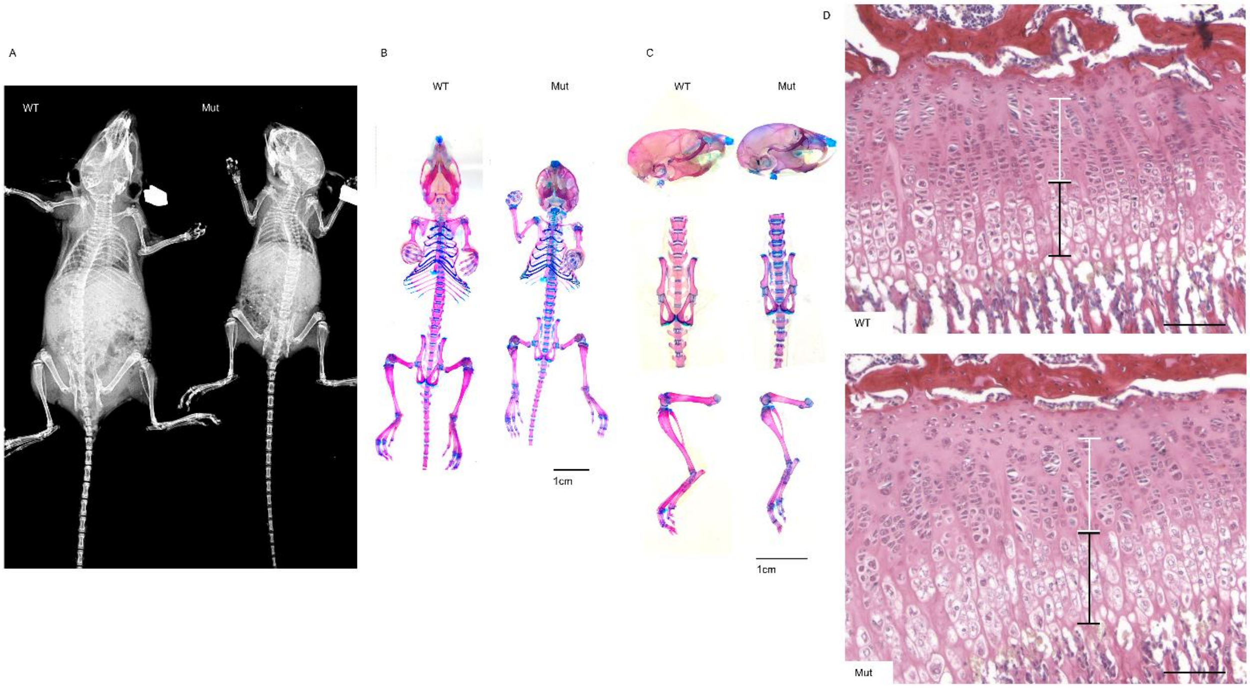

Supplemental Figure 2. Phenotype of mutant mice at weaning. (**A**) Representative AP radiographs demonstrating the smaller size of mutant mice (Mut) relative to an unaffected littermate (WT) but absence of the severe radiographic phenotype observed at 8 weeks. (**B**) Skeletal preparations illustrating the increased Alcian blue staining in the mutant spines. (**C**) Skeletal elements from B, allowing comparison of the WT and mutant mouse craniofacies, spine and lower limbs. (**D**) Picrosirius red stained proximal tibial growth plates illustrating the disorganized proliferative zone chondrocytes in the mutant (bottom) as compared with the WT mouse (top) with relatively normal hypertrophic zones.

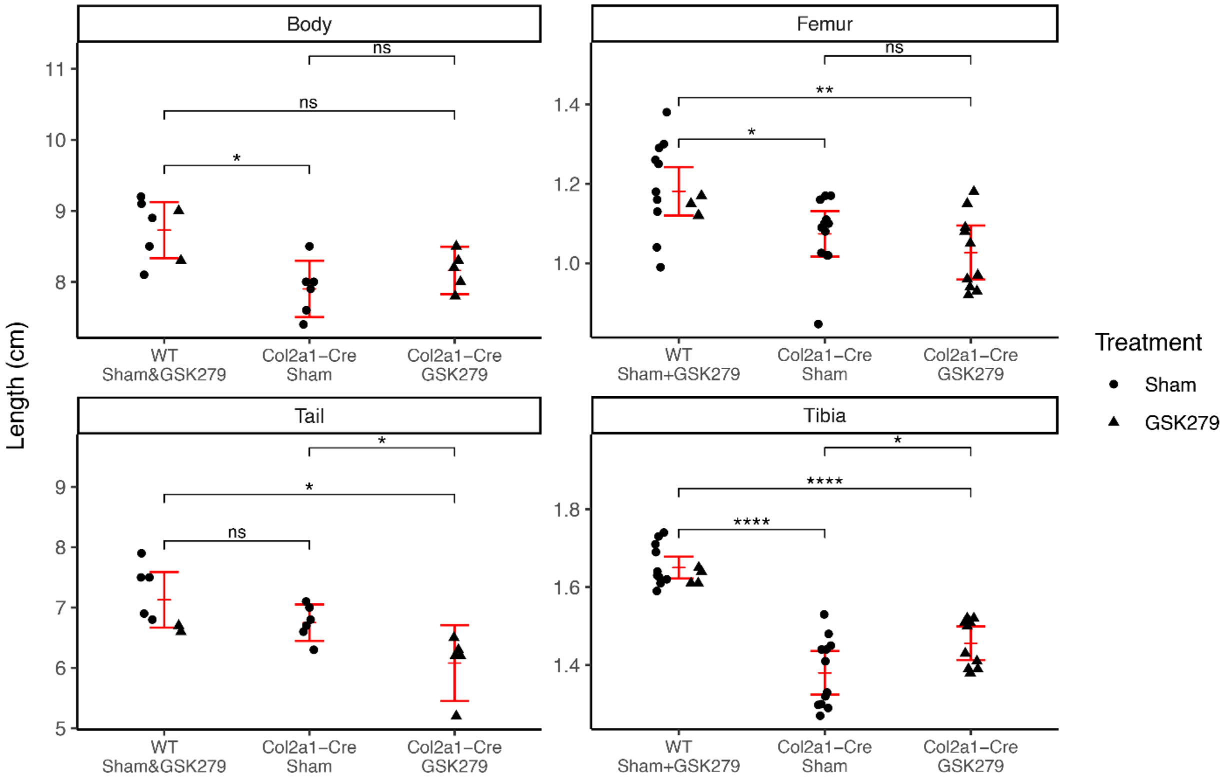

Supplemental Figure 3. Sizes of GSK279-treated *Col2a1-Cre Trpv4* mutant male mice compared with sham-treated mutants. Average body, tail femur and tibia lengths of the WT mice were not significantly affected by inhibitor treatment (shown by brackets on the left side of each panel and labeled ns) and thus combined for comparisons to the sham and inhibitor-treated mutant mouse groups. Note that treatment did not significantly affect the measured parameters. P values were calculated using a Mann-Whitney *U* test. Error bars indicate mean ± SEM. Significance of the differences: ns=p>0.05; *=p<0.05; **=p<0.01; ***=p<0.001; ****=p<0.0001).

Supplemental Table 1. Inhibition of basal and evoked TRPV4 skeletal dysplasia channels by GSK279.

Mutation	Phenotype	FLIPR Ca^{2+} IC_{50}, nM GSK279 (basal)	FLIPR Ca^{2+} IC_{50}, nM GSK279 (evoked)
**$TRPV4^{WT}$**	WT	2.18 ± 0.93 (n=4)	3.25 ± 0.75 (n=4)
**$TRPV4^{R616Q}$**	brachyolmia	4.15 ± 2.26 (n=4)	4.63 ± 0.67 (n=3)
**$TRPV4^{V620I}$**	brachyolmia	2.75 ± 1.13 (n=4)	4.05 ± 1.94 (n=4)
**$TRPV4^{D333G}$**	SMDK	2.63 ± 0.92 (n=3)	9.20 ± 4.21 (n=4)
**$TRPV4^{T89I}$**	MD	2.65 ± 1.22 (n=4)	3.73 ± 1.77 (n=4)
**$TRPV4^{F617L}$**	MD	4.00 ± 1.74 (n=4)	5.83 ± 4.98 (n=4)
**$TRPV4^{P799L}$**	MD	2.38 ± 0.88 (n=4)	3.28 ± 0.11 (n=4)

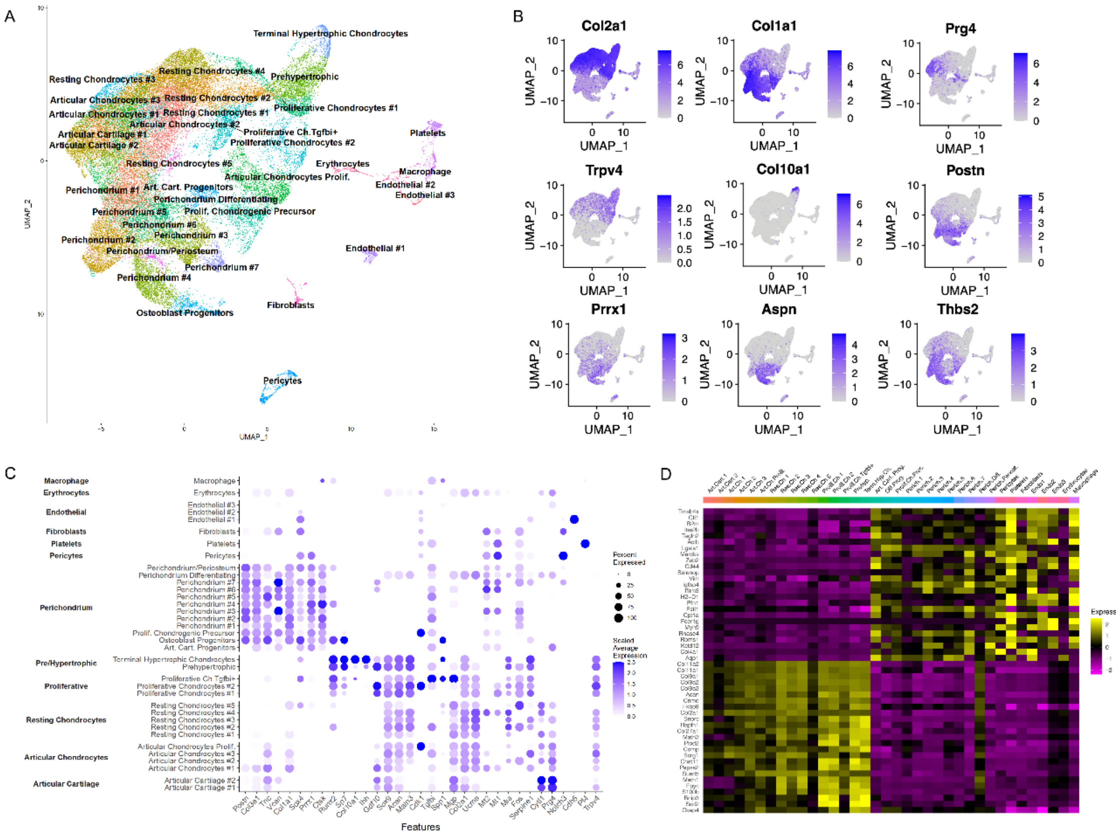

Supplemental Figure 4. Single cell RNA-Seq analysis of P3 WT and *Trpv4* p.R594H growth plates. (**A**) Cell clusters visualized by uniform manifold approximation and projection (UMAP). Colors and labels identify the various cell types (N=3 for WT, N=2 for *Trpv4* p.R594H). (**B**) Feature plots showing the expression of selected chondrocyte markers. (**C**) Dot plot showing the expression of selected markers that highlight gene expression that identifies particular cell types. Dot size represents the percentage of cells expressing a particular gene and color intensity indicates the average expression level for each gene in the cluster. (**D**) Heatmap showing top differentially expressed genes in each cluster.
